# *Fructus Ligustri Lucidi* preserves bone quality through the regulation of gut microbiota diversity, oxidative stress, TMAO and Sirt6 levels in aging mice

**DOI:** 10.18632/aging.102376

**Published:** 2019-11-12

**Authors:** Lin Li, Beibei Chen, Ruyuan Zhu, Rui Li, Yimiao Tian, Chenyue Liu, Qiangqiang Jia, Lili Wang, Jinfa Tang, Dandan Zhao, Fangfang Mo, Yan Liu, Yu Li, Alexander N. Orekhov, Dieter Brömme, Dongwei Zhang, Sihua Gao

**Affiliations:** 1 Diabetes Research Centre, Traditional Chinese Medicine School, Beijing University of Chinese Medicine, Beijing 100029, China; 2 The First Affiliated Hospital of He’nan University of Traditional Chinese Medicine, Zhengzhou 45000, China; 3 The Scientific Research Centre, Traditional Chinese Medicine School, Beijing University of Chinese Medicine, Beijing 100029, China; 4 Department of Histology, Traditional Chinese Medicine School, Beijing University of Chinese Medicine, Beijing 100029, China; 5 Laboratory of Angiopathology, Institute of General Pathology and Pathophysiology, Russian Academy of Medical Sciences, Moscow 125315, Russia; 6 Faculty of Dentistry, University of British Columbia, Vancouver BC V6T 1Z3, Canada

**Keywords:** senile osteoporosis, *fructus ligustri lucidi*, gut microbiota, TMAO, oxidative stress

## Abstract

Gut dysbiosis and oxidative stress may trigger senile osteoporosis. *Fructus Ligustri Lucidi* (FLL) has bone-preserving properties and affects the intestinal microecology. However, the mechanism of the anti-osteoporotic effect of FLL and its link to the gut microbiota remains to be elucidated. Here, we demonstrated that sustained exposure of ICR mice to D-galactose / sodium nitrite for 90 days causes aging-related osteoporosis and reduced cognitive performance. The aging phenotype is also characterized by increased oxidative stress in serum. This is likely triggered by abnormal changes in the gut microbiota population of *Bifidobacterium* and the ratio of *Firmicutes/ Bacteroidetes* that resulted in increased levels of flavin-containing monooxygenase-3 and trimethylamine-N-oxide (TMAO). Moreover, the increased oxidative stress further accelerated aging by increasing tumor necrosis factor-α levels in serum and reducing Sirtuin 6 (Sirt6) expression in long bones, which prompted nuclear factor kappa-B acetylation as well as over-expression and activation of cathepsin K. FLL-treated aging mice revealed a non-osteoporotic bone phenotype and an improvement on the cognitive function. The mechanism underlying these effects may be linked to the regulation of gut microbiota diversity, antioxidant activity, and the levels of TMAO and Sirt6. FLL may represent a potential source for identifying anti-senile osteoporotic drug candidates.

## INTRODUCTION

Osteoporosis is an age-associated skeletal disorder characterized by reduced bone quality and increased fracture risks. The disease primarily affects post-menopausal women, but also has an impact on aging male populations [1]. Although the general incidence rate of fragility fractures is higher in females, osteoporotic men statistically face a greater chance of fracture related mortality [2]. A growing body of evidence suggests that aging may play a central role in the induction of bone loss, regardless of estrogen deficiency [3, 4]. As bone loss primarily occurs in populations aged 30 years and above, the burden of osteoporosis is projected to increase dramatically due to the aging population [5]. Therefore, effective measures are needed for the early diagnosis and prevention of bone fractures. Current treatment options for osteoporosis, mostly anti-resorptive and anti-oxidant medications such as bisphosphonates, denosumab, and vitamin E (VE), are limited in their clinical use because of side effects and lack of evidence for long-term efficacy [5–7]. Recently, plant-based therapies, including traditional Chinese medicine (TCM), have gained growing attention in the management of osteoporosis due to their cost effectiveness, multi-drug targets, and low risk of adverse reactions [8].

Considering that the oxidative stress triggered by reactive oxygen species (ROS) is present throughout life, the sustained accumulation of oxidative damage to cellular lipids, proteins, or DNA in later stages of life will accelerate the process of aging [9]. Over-production of ROS may facilitate osteoclastogenesis through the activation of nuclear factor-kappa B (NF-κB) translocation and acetylation and subsequent cathepsin K (CatK) expression [9, 10]. The upregulation of tumor necrosis factor-alpha (TNF-α) levels further potentiates oxidative stress in aged bones [11]. Meanwhile, the disruption of the redox homeostasis caused by ROS accumulation over natural antioxidant defense mechanisms also impedes osteoblastoegenesis, which results in a relative increase in bone resorption over bone formation and subsequent osteoporosis [10]. The results obtained from preclinical and clinical investigations also support the view that antioxidant treatments may be beneficial for the management of osteoporosis [9]. Additionally, oxidative stress may induce downregulation of the nuclear regulatory enzyme, sirtuin 6 (Sirt6) [12], which is associated with the development of senile osteoporosis through the induction of osteoclastogenesis via the regulation of the NF-κB/CatK signaling pathway [3, 13]. However, major challenges still remain in dissecting the mechanistic details of age-related osteoporosis and effective ways to therapeutically interfere with them.

Emerging evidence suggests that gut dysbiosis, the alteration of mutualistic gut microbial composition, is highly associated with the progression of aging and osteoporosis in rodents and humans [14]. It is of growing interest that the abnormal elevation of trimethylamine-N-oxide (TMAO) levels, one of the gut-flora-derived metabolites, mediates the onset of aging-related cardiovascular disease and cognitive dysfunction partly through increased oxidative stress [15, 16]. During this process, flavin-containing monooxygenase 3 (FMO3) appears to be involved in the aging process through converting bacterial-produced trimethylamine into TMAO [17]. In addition, a dysbiosis in the gut environment may disturb bone remodeling through gut-derived uremic toxins [18].

*Fructus Ligustri Lucidi* (FLL), the dried ripe fruit of *Ligustrum lucidum Ait* and a plant in the olive family, has been used as an anti-aging medicinal plant to treat osteoporosis for more than 1,000 years in TCM [19]. We and others have demonstrated that FLL and its main components exhibit strong antioxidant [10] and anti-aging activities [20], as well as *in vivo* and *in vitro* bone preserving effects [21, 22]. The anti-osteoporotic effects of FLL may be primarily caused by the stimulation of osteogenesis and inhibition of adipogenesis and osteoclastogenesis in aged female, ovariectomized (OVX), and growing rats [22, 23] through: (i) modulating calcium resorption and calcium balance via up-regulating serum 1,25 (OH)_2_D_3_ levels and vitamin D-dependent calcium transport gene expression; (ii) regulating redox homeostasis through the regulation of NADPH oxidase 4 (Nox4)/ROS/NF-κB [24]. In addition, nanoparticle preparations of the FLL compound was demonstrated to improve intestinal microecological disorders and repair intestinal mucosal damage in mice [25, 26]. However, little is known about the regulation of FLL aqueous extracts on the gut microbiota in aging-related osteoporotic mice. Therefore, in the current study, we attempted to investigate whether FLL improves bone quality through the regulation of gut microbiota balance and oxidative stress in D-galactose (D-gal)/ sodium nitrite (NaNO_2_)-induced aging Institute of Cancer Research (ICR) mice, and explore the potential association with Sirt6/NF-κB/CatK signaling pathway.

## RESULTS

### Aging mice demonstrated a deficit in memory and cognitive function which was improved by FLL treatment

**Figure 1:**
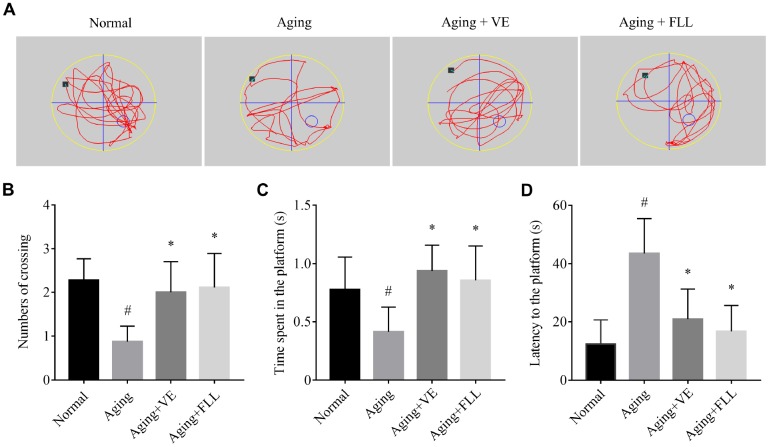
***Fructus Ligustri Lucidi* (FLL) improves memory and cognitive function in aging mice.** (**A**) Representing swimming paths and search strategy of mice in the spatial probe test on the fifth day. (**B**) Numbers of crossing over the hidden platform located in quadrant IV. (**C**) The time spent in the platform. (**D**) Latency to find the platform. [◦: location of target platform (quadrant IV); ■: original location of the mouse (quadrant I)]. Data are presented as mean ± SD. # compared with the normal group. * compared with the aging model group. p < 0.05 was considered statistical difference, n = 9.

As shown in Figure 1, mice in the aging (Aging) group exhibited ~62% lower crossing numbers (Figure 1B) and ~47% lower time spent in the platform area (Figure 1C), along with a ~2-fold higher latency to reach the putative platform area (Figure 1D) when compared to those in the normal non-aged control group (Normal) (*p* < 0.05), suggesting that the D-gal/ NaNO_2_ -induced mice exhibited an aging phenotype. Intriguingly, administration of VE and FLL to aging mice significantly prevented the memory loss by normalizing the number of crossings, time spent in the platform area, and latency to reach the platform area, indicating that FLL may have an anti-aging capacity.

### Aging mice showed a deterioration in bone microarchitecture and reduction in biomechanical properties while FLL demonstrated preservation capabilities

**Figure 2:**
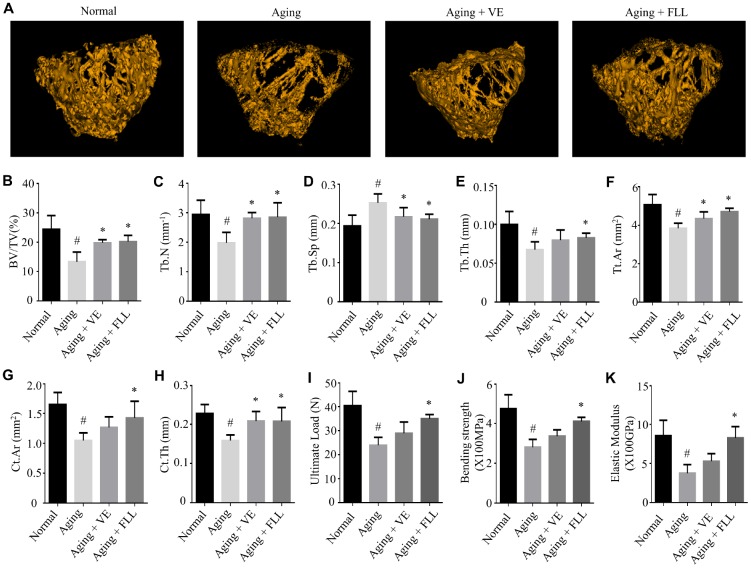
***Fructus Ligustri Lucidi* (FLL) preserves the bone microarchitecture and bone strength in the femurs of aging mice.** Representative 3D reconstructions of trabecular images in the femoral metaphysis by micro(μ)-CT (**A**). μCT-derived quantification data of femoral metaphysis including BV/TV (%, **B**), Tb.N (1/mm) (**C**), Tb.Sp (mm) (**D**), Tb.Th (mm) (**E**), Tt.Ar (mm^2^) (**F**), Ct.Ar (mm^2^) (**G**), Ct.Th (mm, **H**). Three-point bending examination results including ultimate load (**I**), bending strength (X100MPa, **J**), and elastic modulus (X100GPa, **K**) in the femurs. Data are presented as mean ± SD. # compared with the normal group. * compared with the aging model group. *p* < 0.05 was considered statistical difference, n = 8.

To examine whether the bone microstructure was altered in the femurs of aging mice and whether FLL could protect it, micro–computed tomography (μCT) was employed to analyze the bone-morphometric parameters in the femurs of the different groups of mice. As shown in Figure 2A–2H, the administration of D-gal and NaNO_2_ induced a significant loss of femoral bone, characterized by decreased bone volume (BV)/total volume (TV), trabecular numbers (Tb.N), trabecular thickness (Tb.Th), total cross-sectional area (Tt.Ar), cortical bone area (Ct.Ar), and cortical thickness (Ct.Th), while it increased trabecular separation (Tb.Sp) (*p* < 0.05). Interestingly, FLL treatment significantly halted bone loss in the femurs of aging mice (*p* < 0.05). In contrast, VE treatment only partly inhibited the trend of changes in the above-mentioned parameters.

To further evaluate if these alterations will affect bone biomechanical properties, mice femurs were subjected to the three-point bending assay. As shown in Figure 2I–2K, the ultimate load, bending strength, and elastic modulus were ~41%, ~41%, and ~56% lower, respectively, in the mice femurs of the Aging group than those in the normal controls (*p* < 0.05). However, treatment of aging mice with FLL prevented a decrease in the above-mentioned bone strength parameters by ~69%, ~67%, and ~94%, respectively (*p* < 0.05). Surprisingly, VE treatment did not prevent the reduction of bone strength in aging mice. These results suggest that the administration of D-gal and NaNO_2_ to mice negatively affected the microstructure and strength of the analyzed femurs. In contrast, FLL treatment maintained the bone microarchitecture parameters and strength near to the levels of the Normal control group during aging.

Next, we determined bone quality in the aging mice. As shown in Table 1, Fourier-transform infrared spectroscopy (FTIR) results indicated that the relative ratio of mineral to matrix and acid phosphate substitution were significantly reduced, while the collagen maturity and crystallinity were significantly increased in the mice femurs of the Aging group when compared to parameters in the Normal controls (*p* < 0.05). Treatment of aging mice with FLL or VE evidently maintained the trend of the measured FTIR parameters in the femurs compared with those of the vehicle-treated aging animals (*p* < 0.05).

**Table 1:** Bone material properties determined by FTIR.

**Group**	**Mineral/Matrix peak area ratio**	**Collagen maturity peak high ratio**	**Crystalinity**	**Acid phosphate substitution**
**Normal**	3.6737±0.2675	1.0828±0.0203	1.0148±0.0075	0.8792±0.0244
**Aging**	3.2966±0.1582^#^	1.1150±0.0146^#^	1.0247±0.0042^#^	0.8504±0.0169 ^#^
**Aging+VE**	3.3220±0.2917	1.0653±0.0232^*^	1.0105±0.0045^*^	0.8865±0.0215^*^
**Aging+FLL**	3.5700±0.1642^*^	1.0747±0.0279^*^	1.0134±0.0065^*^	0.8888±0.0265 ^*^

Data are presented as mean ± SD. FTIR: Fourier-transform infrared spectroscopy. ^#^ compared with the Normal group. * compared with the Aging group, p < 0.05 was considered statistical difference, n=10.

**Figure 3:**
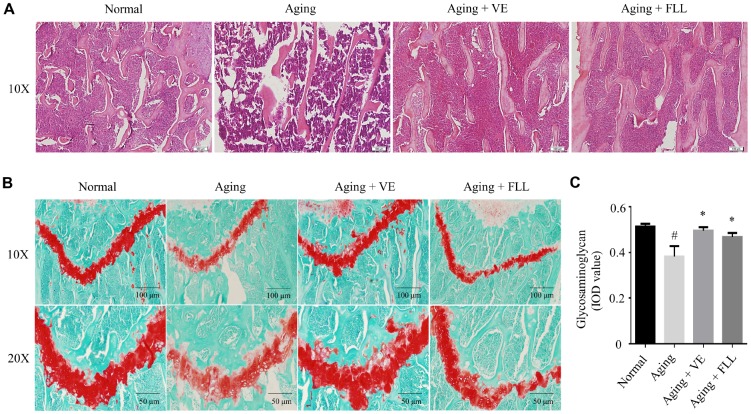
***Fructus Ligustri Lucidi* (FLL) preserves bone histomorphological features in aging mice.** Representative images of H&E staining in the femoral metaphysis of different groups of mice (**A**). Safranin O/Fast green staining (**B**) and image analyses (**C**) in the femoral metaphysis of different groups of mice. Data are presented as mean ± SD. # compared with the normal group. * compared with the aging model group. p < 0.05 was considered statistical difference, n=8.

These finding were also supported by various histological analyses. As shown in Figure 3A, Hematoxylin and eosin (H&E) staining revealed that the trabecular bone was composed of a dense and regular fiber meshwork in the distal femurs of the Normal group of mice. However, the trabecular bone lost the regular mesh architecture, and turned thinner and irregular in the distal femurs of the vehicle-treated aging control mice. Interestingly, trabecular bones retained their structures in the mice femurs of VE (Aging+VE), and FLL group (Aging+FLL) groups.

To further observe the pathological alterations, safranin O/fast green staining was used to detect alterations of the epiphyseal structure in the femurs of aging mice. As shown in Figure 3B and 3C, the thickness of the epiphyseal growth plate decreased by ~25% in the distal femurs of the vehicle-treated aging group of mice when compared to that of vehicle-treated normal controls (*p* < 0.05). Intriguingly, administration of FLL or VE to aging mice for 65 days increased the thickness of cartilage in the growth plate by ~24% and ~32%, respectively, as compared to vehicle-treated aging mice (*p* < 0.05).

In addition, the concentration of C-terminal telopeptide of type 1 collagen (CTx-1), a circulating CatK-generated bone resorption marker, increased ~2-fold (*p* < 0.05), while the levels of procollagen type 1 N-terminal propeptide (P1NP), a circulating bone formation marker, decreased by ~19% (*p* < 0.05) in the mice of the aging control group compared with the normal controls (Table 2). The administration of FLL to aging mice for 65 days also significantly prevented the bone resorption-formation imbalance (*p* < 0.05). However, VE only increased serum levels of CTx-1 in aging mice compared to vehicle-treated aging ones. These findings suggest that FLL may improve bone healthy remodeling in aging mice.

**Table 2:** The serum levels of TAC, MDA, 8-OHdG, GSH/GSSG, P1NP, and CTx-1 in mice.

**Group**	**TAC (mM)**	**MDA(μM)**	**8-OHdG (ng/L)**	**GSH/GSSG**	**P1NP(ng/mL)**	**CTx-1 (ng/mL)**
**Normal**	1.82±0.50	5.90±1.40	7.74±2.70	6.48±1.57	5.28±0.32	5.13±1.47
**Aging**	1.15±0.14^#^	9.35±0.88^#^	29.31±3.01^#^	1.71±0.65^#^	4.29±0.38^#^	17.15±2.65^#^
**Aging+VE**	1.50±0.08^*^	7.30±0.79^*^	12.20±4.19^*^	4.19±0.72^*^	4.61±0.21	8.96±1.67^*^
**Aging+FLL**	1.54±0.06^*^	7.55±1.26^*^	11.34±3.63^*^	2.92±0.80^*^	4.75±0.32^*^	10.52±2.36^*^

Data are presented as mean ± SD. 8-OH-dG: 8-hydroxy-2′-deoxyguanosin, CTx-1: C-terminal telopeptide of type 1 collagen, GSH: Glutathione (reduced), GSSG: Glutathione (Oxidized), MDA: Malondialdehyde, TAC: total antioxidant capacity, P1NP, Procollagen type 1 N-terminal propeptide. # compared with the Normal group. * compared with the Aging group, p < 0.05 was considered statistical difference, n=8.

### FLL inhibited oxidative stress in aging mice

**Figure 4:**
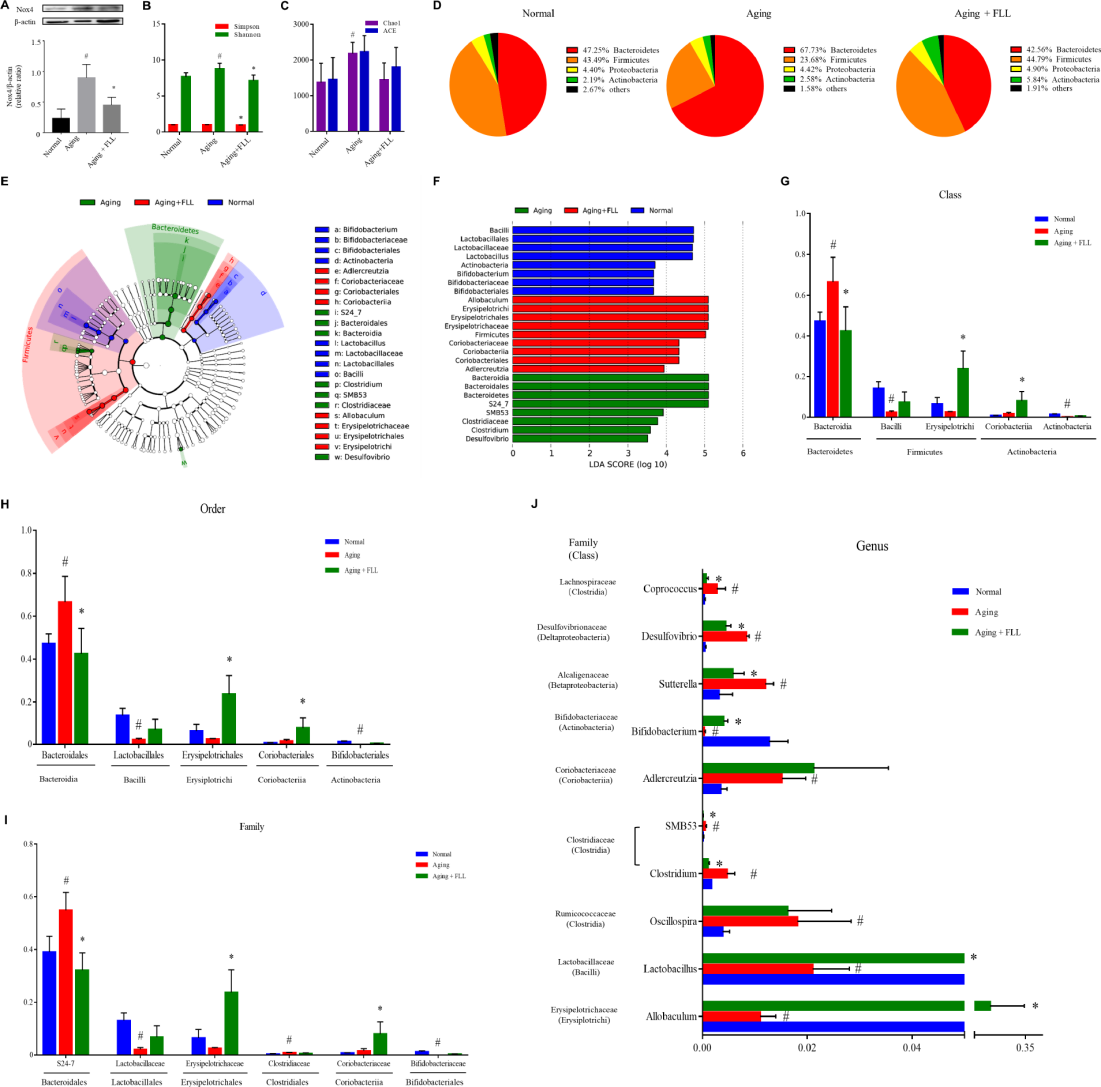
***Fructus Ligustri Lucidi* (FLL) decreases Nox4 expression and modulates the impaired gut microbiota in aging mice.** (**A**) The representative western blot image and their analysis showed tibias Nox4 expression. (**B**) Shannon and Simpson diversity indices. (**C**) Chao 1 and ACE diversity indices. (**D**) Relative abundances of bacterial operational taxonomy units (OTUs) at the phylum level. LEfSe cladograms (from kingdom to genus) (**E**) and Linear discriminant analysis (LDA)-derived histogram of differentially abundant taxa (**F**). Relative abundances of major bacterial OTUs in class (**G**), order (**H**), family (**I**), and genus (**J**) level. Data are presented as mean ± SD. # compared with the normal group. * compared with the aging model group. *p* < 0.05 was considered statistical difference, n=5.

Oxidation-antioxidant imbalance leading to oxidative stress is closely related to aging, which damages biological macromolecules such as DNA, proteins, lipids, and organelles in cells. Next, we assessed the effect of FLL on serum redox markers in aging mice. 8-hydroxy-2′-deoxyguanosine (8-OH-dG) is an important marker for the degree of oxidative damage in genomic DNA in the nucleus. Malondialdehyde (MDA) can reflect the production of lipid peroxides. Total antioxidant capacity (TAC) is a comprehensive indicator used to measure the functional status of the body's antioxidant system. The ratio of reduced glutathione/oxidized glutathione disulfide (GSH/GSSG) reflects the redox balance status. Nox4 is regarded as one of the most main sources for ROS generation [8]. As shown in Table 2, the levels of TAC and GSH/GSSG decreased by ~37% and ~73%, and the levels of MDA and 8-OH-dG increased by ~58% and ~2.8-fold, respectively, in the serum of vehicle-treated aging mice relative to the vehicle-treated Normal controls (*p* < 0.05). Interestingly, the administration of VE and FLL to aging mice attenuated the increase of MDA in serum by ~59% and ~52 %, and the increase of 8-OH-dG in serum by ~81% and ~85%. Moreover, FLL reduced the decrease of TAC in serum by ~52% and ~58%, and the decrease in serum GSH/GSSG ratios by ~52 % and ~25 %, respectively (*p* < 0.05). In addition, FLL treatment also reduced the increased Nox4 expression in the tibias of aging mice (Figure 4A). These findings suggest that FLL may improve antioxidant activity through downregulating the levels of MDA, 8-OH-dG, and Nox4, as well as by upregulating the levels of TAC and the ratio of GSH/GSSG in aging mice.

### FLL modulated gut microbiota composition in aging mice

Subsequently, we examined the influence of D-gal and NaNO_2_ on the intestinal microbial communities in aging mice and the involvement of FLL in this process. The alpha diversity analysis, including the Shannon diversity index (Figure 4B) and Chao1 index (Figure 4C), for gut microbiomes indicated that the overall microbial assortments in the aging mice control group increased when compared to those of the normal controls (*p* < 0.05); However, FLL treatment significantly prevent the increase of Shannon and Simpson diversity indexes (*p* < 0.05), and has the trend to prevent the increase of Chao1 index. The proportion of the phyla *Firmicutes* was decreased, while those of *Bacteroidetes* and *Actinobacteria* was increased in the aging control group relative to the normal controls (Figure 4D). Intriguingly, in the Aging+FLL group, the ratio of *Firmicutes* to *Bacteroidetes* was comparable to normal control levels; However, the proportion of *Actinobacteria* was notably higher than in the other groups (*p* < 0.05). To further characterize the specific gut microbiota compositional differences, we focused on the key community members derived from the Linear discriminant analysis effect size (LEfSe) analysis (Figure 4E and 4F). The LEfSe classification hierarchical tree cladogram from the inner ring kingdom to the outer ring genus demonstrated that the class *Bacteroidia* and family *S24_7* were the most discriminant microbial taxa in the vehicle-treated aging mice, while the class *Erysipelotrichi* and *Coriobacteriia* were the most critical bacterial taxa in the Aging+FLL group. Furthermore, to compare the relative contents of intestinal bacterial taxa in response to D-gal and NaNO_2_ stimulation and FLL intervention, the altered bacteria were selected in accordance with relative Operational Taxonomic Unit (OTU) abundance. A total of 5 classes (*Bacteroidia*, *Bacilli*, *Erysipelotrichi*, *Coriobacteriia*, and *Actinobacteria*), 5 orders (*Bacteroidales*, *Erysipelotrichales*, *Lactobacillales*, *Coriobacteriales*, and *Bifidobacteriales*), 6 families (*S24-7*, *Lactobacillaceae*, *Erysipelotrichaceae*, *Coriobacteriaceae*, *Bifidobacteriaceae* and *Clostridiaceae*), and 10 genera differed noticeably among the test groups (Figure 4G–4J). At the genus levels, 10 genera showed significant differences between the groups. Specifically, D-gal and NaNO_2_ stimulation triggered an increase in *SMB53*, *Clostridium*, *Adlercreutzia*, *Sutterella*, *Oscillospira*, *Coprococcus*, and *Desulfovibrio*, and a decrease in *Allobaculum*, *Lactobacillus*, and *Bifidobacterium*; whereas FLL could prevent the above alterations with the exception of *Aldercreutzia* and *Oscillospira.*

### FLL decreased the circulating TMAO levels in aging mice

**Figure 5:**
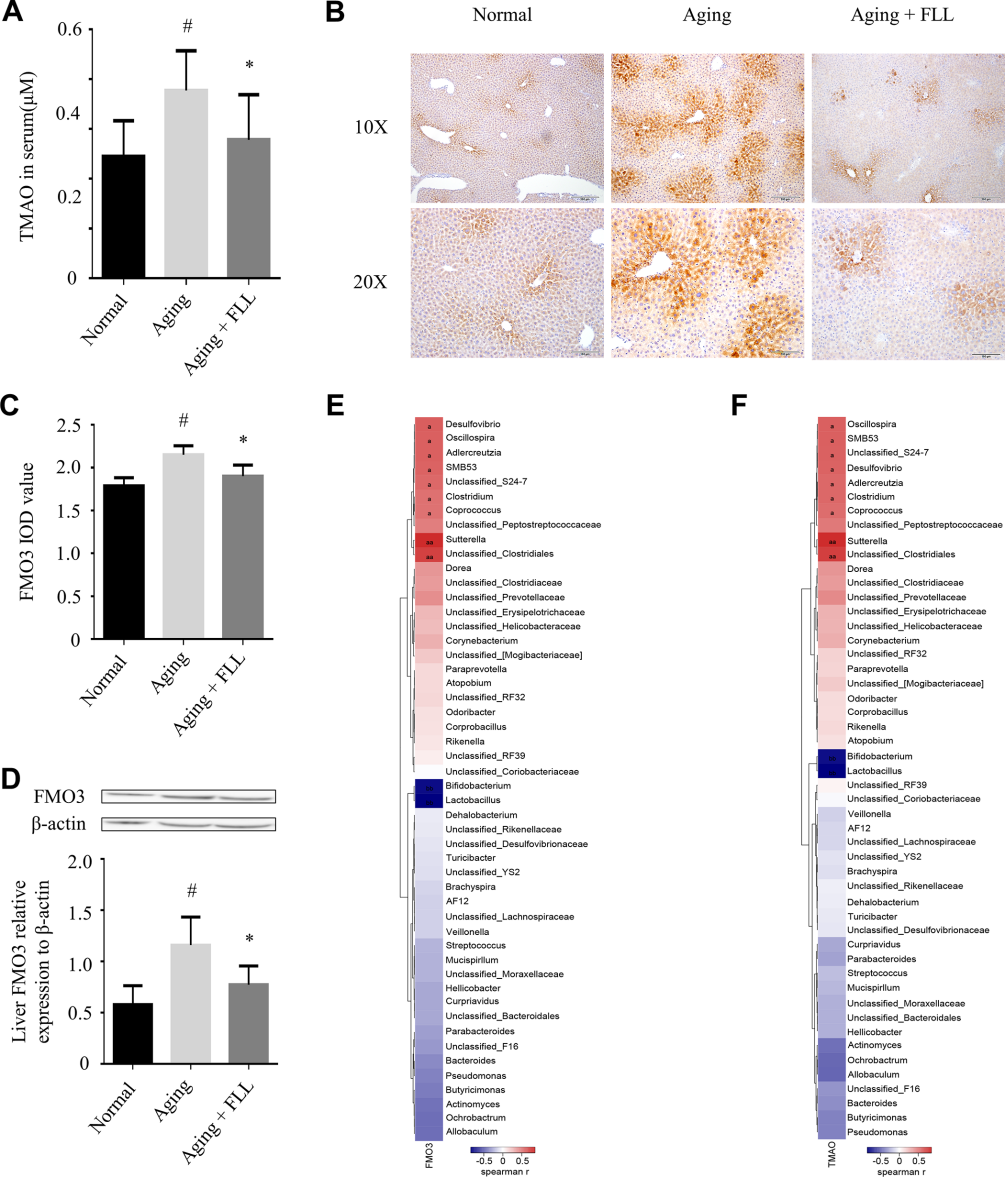
***Fructus Ligustri Lucidi* (FLL) decreases the expression of FMO3 and the levels of circulating TMAO in aging mice.** (**A**) The circulating TMAO levels quantified by LC/MS/MS. The expression levels of FMO3 were analyzed by immunohistochemical staining (**B**–**C**) and western blot (**D**) in the livers. (**E**–**F**) Correlation heat map indicating the association between the specific microbiota taxonomic genera and FMO3 expressions (**E**) and TMAO levels (**F**). Red indicates a positive association, blue denotes a negative association, and white manifests no association. *a* denotes a significant false discovery rate (FDR)-adjusted positive correlation at p values of < 0.05, and *aa* denotes a significant FDR-adjusted positive correlation at p values of < 0.01. *b* denotes a significant FDR-adjusted negative correlation at p values of < 0.05, and *bb* denotes a significant FDR-adjusted negative correlation at P values of < 0.01. Data are presented as mean ± SD. # compared with the normal group. * compared with the aging model group. *p* < 0.05 was considered statistical difference, n=8.

TMAO, one of the gut microbiota-derived metabolites, is implicated in the development of aging-related diseases [16]. As shown in Figure 5A, the liquid chromatography tandem-mass spectrometry (LC-MS/MS) assay shows that the serum TMAO levels were significantly higher in mice from the aging control group (0.38 ± 0.08 μM) than in those from the normal controls (0.24 ± 0.07 μM) (*p* < 0.05), and administration of FLL to aging mice for 65 days significantly reduced serum TMAO levels (0.28 ± 0.09 μM) (*p* < 0.05). The results indicate that FLL intervention could stabilize the levels of circulating TMAO in aging mice.

Liver FMO3 is essential for the generation of TMAO [27]. Therefore, we investigated whether FMO3 was altered in the aging mice, as well as the effect of FLL treatment on FMO3 levels using immunohistochemistry (IHC) staining and western blot analysis. As shown in Figure 5B–5D, we found that FMO3 expression levels were significantly elevated in the livers of the aging control group when compared to those of the normal controls (*p* < 0.05). Interestingly, FLL treatment significantly reduced the FMO3 expression levels in the livers of aging mice when compared to those of the vehicle-treated ones (*p* < 0.05).

Furthermore, correlation heat map analysis was applied to evaluate the specific gut microbiota taxonomic genera associated with the production of FMO3 or TMAO between the mice of Normal, Aging and Aging+FLL group as shown in Figure 5E and 5F. After false discovery rate (FDR) adjustment for multiple comparisons, we found that species of several bacterial taxa (*Sutterella*, *Unclassified_Clostridiales*, *Corpococcus*, *Clostridium*, *Unclassified_S24-7*, *SMB53*, *Aldercreutzia*, *Oscillospira*, *Desulfovibrio*, *Bifidobacterium*, and *Lactobacillus*) were significantly associated with liver FMO3 and/or serum TMAO levels (*p* < 0.05).

### FLL increased Sirt6 expression in tibias and femurs of aging mice

**Figure 6:**
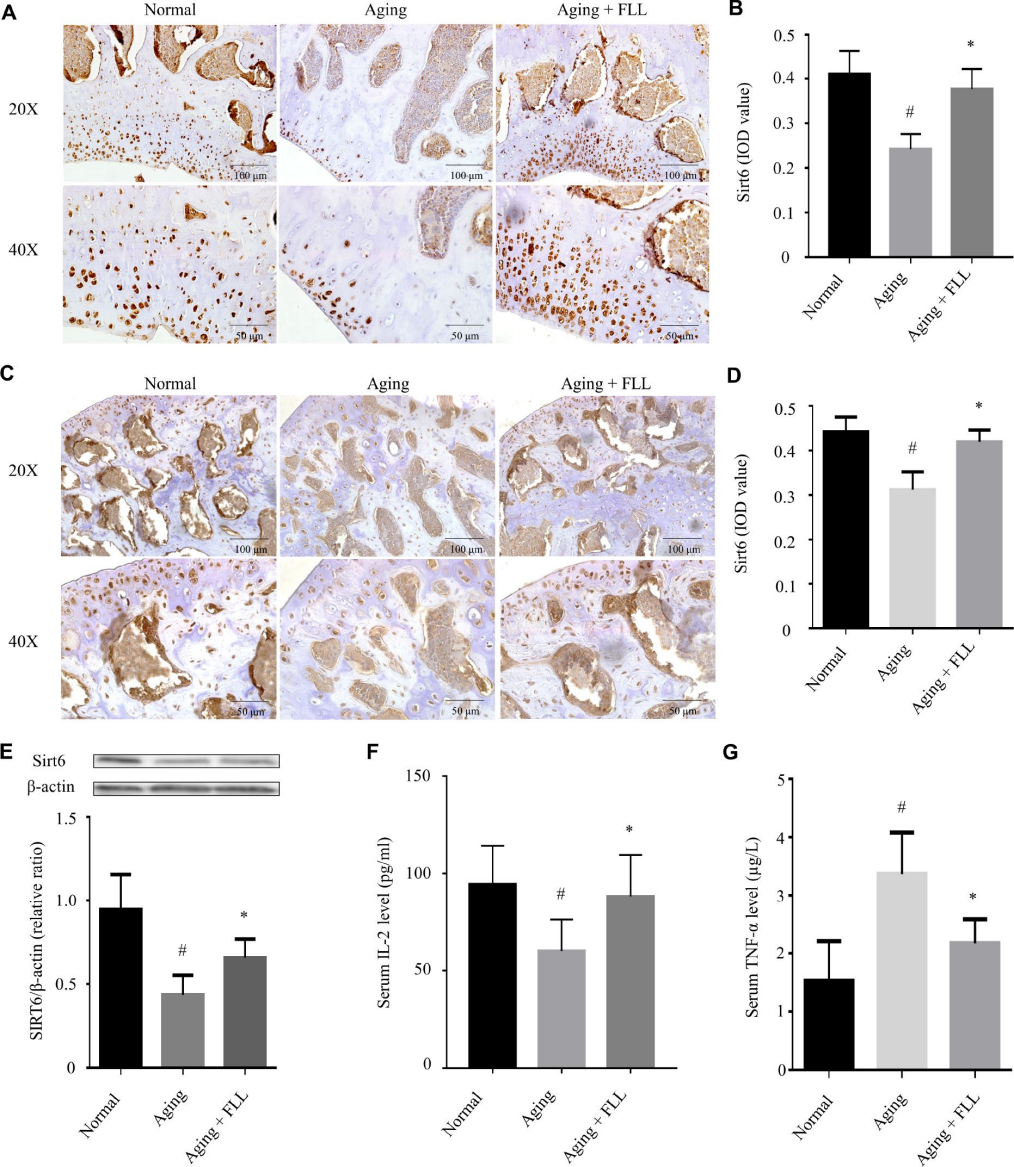
***Fructus Ligustri Lucidi* (FLL) increases tibial and femoral Sirt6 expression and regulates serum levels of TNF-α and IL-2 in aging mice.** Immunohistochemical staining and western blot showed Sirt6 expression (original magnification, ×200, ×400) in the femoral metaphysis (**A**–**B**) and femoral heads (**C**–**D**), and in the tibias (**E**) of different groups of mice. Serum levels of IL-2 (**F**) and TNF-α (**G**). Data are presented as mean ± SD. # compared with the normal group. * compared with the aging model group. *p* < 0.05 was considered statistical difference, n=8.

Next, we investigated the effect of FLL on Sirt6 expression in the femurs and tibias of aging mice using western blot and IHC. As shown in Figure 6A–6D, the expression levels of Sirt6 in distal femurs and femoral heads of the vehicle-treated aging group were ~41% and ~30%, respectively, lower than those of vehicle-treated normal controls (*p* < 0.05). Interestingly, administration of FLL to aging mice significantly increased Sirt6 expression levels by ~44% in the femurs in comparison with those of vehicle-treated aging controls (*p* < 0.05). Similar results were also obtained from the tibias tested by western blot (Figure 6E).

It has been shown that a decline in interleukin-2 (IL-2) production is paralleled with aging and age-related diseases [28]. Therefore, we decided to examine serum IL-2 levels in the mice as well. As shown in Figure 6F, injection of D-gal and NaNO_2_ into the ICR mice induced a decrease in IL-2 production by ~37% compared to the vehicle-treated normal controls (*p* < 0.05). Interestingly, FLL treatment increased serum IL-2 production by ~82% in aging mice (*p* < 0.05).

Prompted by the evidence that there were increased levels of tumor necrosis factor-alpha (TNF-α) in aged bones and that oxidative stress potentiates TNF-α production, which has been known to further potentiate oxidative stress [11], we determined serum TNF-α levels in the normal and aging groups. As expected, injection of D-gal and NaNO_2_ into the ICR mice for 3 months led to a 1.2-fold increase in TNF-α production when compared to vehicle-treated normal controls (*p* < 0.05). In contrast, FLL treatment reduced serum TNF-α production to near-normal level in aging mice (*p* < 0.05).

### FLL inhibited NF-κB activation in tibias and femurs of aging mice

**Figure 7:**
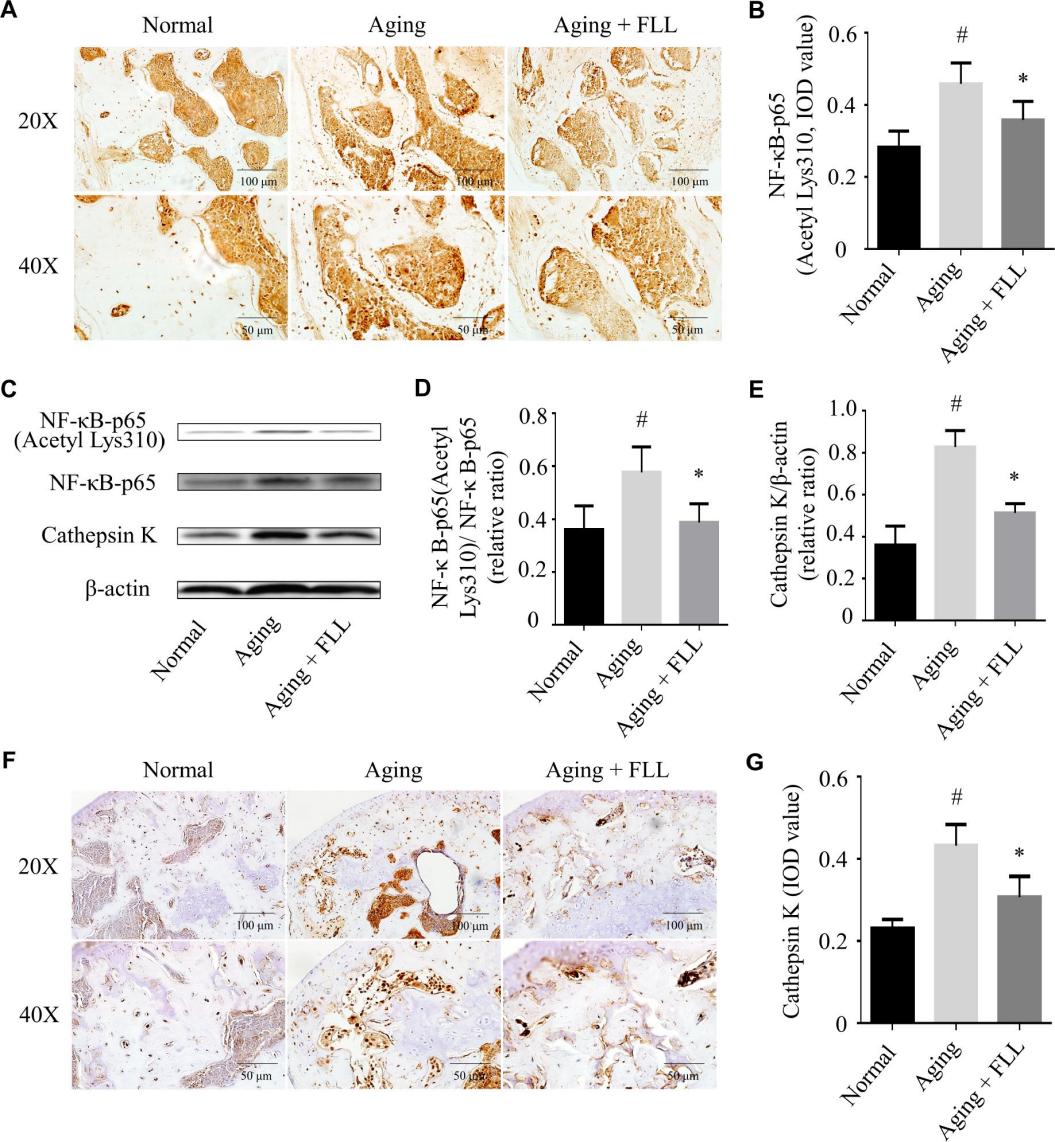
***Fructus Ligustri Lucidi* (FLL) inhibits NF-κB activation and CatK expression in the tibias and femurs of aging mice.** The representative images of immunohistochemical staining (original magnification, ×200, ×400) and western blot, and their analyses showed the acetylation of NF-κB-p65 (**A**–**B**), relative expression of NF-κB-p65 acetylation to NF-κB-p65 (**C**–**D**) and CatK (**C**, **E**–**G**) in the femurs and tibias. Data are presented as mean ± SD. # compared with the normal group. * compared with the aging model group. *p* < 0.05 was considered statistical difference, n=6.

Exposure to oxidative stress or deficiency in Sirt6 expression may lead to NF-κB activation, which further induces bone destruction [9, 13]. Therefore, we determined whether FLL affected NF-κB activation in aging mice. As shown in Figure 7A and 7B, NF-κB-p65 (Acetyl Lys310) expression levels increased by ~64% in the femurs of vehicle-treated aging mice relative to those of vehicle-treated normal controls (*p* < 0.05). After the mice were treated with FLL for 65 days, the staining intensity of NF-κB-p65 (Acetyl Lys310) was decreased by ~23% in the femurs when compared with the vehicle-treated aging controls (*p* < 0.05), indicating that FLL treatment reduced NF-κB-p65 acetylation in the femurs of aging mice. Similarly, western blot results revealed that administration of FLL to aging mice for 65 days exhibited a marked reduction (~46%) in the relative expression ratio of NF-κB-p65 acetylation to NF-κB-p65 in the tibias when compared with vehicle-treated aging ones (*p* < 0.05). These results show that FLL may inhibit osteoclastogenesis through the regulation of NF-κB-p65 activation in aging mice.

### FLL decreased CatK expression in tibias and femurs of aging mice

CatK activity is closely related to the bone resorbing activity of osteoclasts with its ability to break down triple helical collagen [29]. As expected, the results from western blot analysis (Figure 7C and 7E) and IHC staining (Figure 7F and 7G) indicated that CatK expression was significantly increased in the proximal femur trabecular area of vehicle-treated aging animals when compared to those of vehicle-treated normal ones (*p* < 0.05). FLL intervention decreased CatK expression by 28% in aging mice in comparison with vehicle treatment (*p* < 0.05). The results indicate that FLL possesses the ability to suppress bone resorption through inhibiting CatK expression in aging mice.

## DISCUSSION

Chronic exposure of mice to D-gal and NaNO_2_ triggers the acceleration of natural senescence due to increased oxidative stress [30]. This will further lead to the induction of osteoporosis and the increased risk of bone fractures. Indeed, the present work demonstrated for the first time that the administration of D-gal and NaNO_2_ into ICR mice induced a reduction in bone integrity and strength. This aging mouse model is associated with aggravated oxidative stress verified by decreased levels of TAC and GSH/GSSG, and increased levels of MDA and 8-OH-dG in serum. In addition, we found that the increased oxidative stress is associated with an abnormal gut microbiota and consequently increased levels of FMO3 and TMAO. The increased oxidative stress further accelerated aging by decreasing IL-2 levels and increasing TNF-α levels in the serum and reducing Sirt6 expression in the tibias and femurs, which triggered NF-κB activation and CatK expression. Importantly, we found that FLL treatment could prevent aging-induced osteoporosis through a reversal of the changes discussed above.

Currently, a variety of active ingredients – mainly flavonoids, terpenoids, phenylethanoid glycosides, phospholipids, polysaccharides, and volatile components – have been identified in FLL [24]. In addition, earlier investigations by our group and other laboratories have shown that the aqueous extract of FLL primarily contains oleanolic acid, ursolic acid, salidroside, ligustroflavon, acteoside, specnuezhenide, oleuropein acid, nuezhenide, neuzhengalaside, nuezhenoside, tyrosol, tyrosyl acetate, hydroxytyrosol, G13, oleoside dimethyl ester, and oleoside-7-ethyl-11-methyl ester [10, 31, 32]. It is likely that some of the identified compounds in the FLL aqueous extract may represent the active constituents that are beneficial against aging.

In the present study, we demonstrated that the chronic injection of D-gal and NaNO_2_ into ICR mice resulted in increased oxidative stress and accelerated aging, which subsequently leads to reduced bone quality including a loss of bone strength. Indeed, several investigators have also demonstrated that the chronic administration of D-gal and NaNO_2_ to mice produces aging phenotypes by disrupting the redox homeostasis and increasing cognitive dysfunction [30, 33, 34]. The over-production of ROS may trigger osteoporosis by favoring osteoclastogenesis and inhibiting osteoblastogenesis [11, 35]. We and others have demonstrated that FLL aqueous extract contains various antioxidants, such as tyrosol, tyrosyl acetate, hydroxytyrosol, and salidroside, which may counteract the action of oxidants and thus contribute to healthy bone remodeling [12, 32]. Therefore, it is reasonable to suggest that FLL may protect against aging induced osteoporosis by its antioxidant properties.

In the present study, we demonstrated an imbalance in the intestinal microbiota and an increase in serum TMAO and liver FMO3 levels in aging mice, induced by D-gal and NaNO_2_. These findings prompted us to search for a mechanism linking the gut microbiota to aging. It has been shown that a reduction in *Bifidobacterium* and *Lactobacillus* [36–39], and an increase of the *Clostridium, SMB53, Oscillospira, Sutterella, Desulfovibrio* and *Coprococcus* populations in the gut microbiota of aging mice may lead to high circulating trimethylamine and TMAO levels, which consequently leads to increased oxidative stress [27, 40–44]. In addition, the increased liver FMO3 expression contributes to the elevation of TMAO levels [45]. Indeed, we demonstrated that gut dysbiosis is associated with the alteration of FMO3 expression and TMAO production by FDR adjustment analysis. Moreover, gut dysbiosis and its resultant TMAO overproduction may lead to cognition impairment [17, 46] through inhibition of Sirt6 expression via ROS overproduction [12, 47, 48]. Interestingly, in our present study we found that FLL may prevent the increase in the amount of pro-aging gut microbiota mentioned above. This is in agreement with the previous findings that the major ingredients in FLL, such as oleanolic acid, ursolic acid, and salidroside, could modulate microbial diversity in neonatal piglets [49], as well as the microbiota phyla abundance in male Drosophila melanogaster [50] and in furan-induced liver injury-affected mice [51]. Collectively, these observations may support the view that FLL also exerts anti-aging effect through lowering oxidative stress via the regulation of FMO3 and TMAO signaling driven by gut microbiota.

The current study discovered that FLL effectively improves bone microstructure, its material constituents, and bone strength in D-gal and NaNO_2_ treated mice. Previously, we have shown that FLL improves the bone microstructure and increases cortical bone thickness in OVX rats [10]. Given that aging is associated with a significantly higher risk of bone loss and fractures [52], we investigated the effect of FLL on bone alterations during aging. We found that FLL treatment increased bone volume and improved the overall quality of trabecular bone in aging mice, as reflected in the various bone morphometric parameters. Moreover, we demonstrated that 65 days of administration of FLL to aging mice decreased the collagen maturity and crystallinity, as well as increased the mineral-to-matrix ratio and acid phosphate substitution. Accumulating evidence suggests that collagen maturity and crystallinity were related to increasing bone age [53, 54]. Moreover, increased acid phosphate substitution and mineral-to-matrix ratio indicate a more recently formed bone [53]. Thus, we inferred that FLL might delay the bone matrix aging process. In addition, deterioration of bone material composition and microstructure leads to the decreased bone strength [55]. Intriguingly, we illustrated that FLL treatment improved ultimate load, bending strength, and elastic modulus. This is consistent with the literature, which indicates FLL aqueous extract or its major constituents (oleuropein acid and ursolic acid) could improve bone micro-architectural and mechanical properties in aged-OVX or growing female rats [25, 56]. Together, these findings suggest that FLL may preserve bone quality and strength in aging mice.

In the current study, we demonstrated that serum TNF-α levels and bone CatK expression were increased in aging mice and that FLL had a significant therapeutic effect on reversing some of the aging parameters. Increased TNF-α levels in aged bones potentiate oxidative stress and NF-κB activation, which subsequently aggravates bone loss through the inhibition of bone formation and promotion of bone resorption via increasing CatK expression [11]. In addition, overexpression of Sirt6 may suppress TNF-α-mediated NF-κB activation [57]. Interestingly, we demonstrated that increased expression levels of Sirt6 and reduced expression levels of acetyl-NF-κB p65(Lys310) in the femurs and tibias of aging mice. As mentioned above, intestinal flora dysbiosis triggers an elevation of circulating TMAO and subsequent oxidative stress [27, 38, 41], which further inhibits Sirt6 expression [12, 47]. Sirt6 deficiency may cause senile osteoporosis through the promotion of osteoclastogenesis and inhibition of osteoblastogenesis in mice [58] via activation of NF-κB/CatK signaling pathway [3, 14]. In the current study, we demonstrated that FLL could decrease serum TMAO levels, lower oxidative stress, and improve antioxidant enzyme activity. Notably, ursolic acid and hydroxytyrosol, both key active ingredients in the FLL extract, have also been demonstrated to enhance hypothalamus Sirt6 expression in the aged mice [59]. Similarly, we previously demonstrated that FLL aqueous extract was able to inhibit bone loss in OVX rats through the inhibition of NF-κB activation and CatK expression [10]. Furthermore, FLL aqueous extract has been shown to inhibit bone loss and adipogenesis in aged OVX rats and mesenchymal stem cells [23]. In conjunction with these observations, it is conceivable that FLL may prevent bone loss through the regulation of the TMAO/ROS/Sirt6 /NF-κB/CatK signaling pathways in aging mice.

It should be noted that our study has several limitations. Although we demonstrated that FLL preserves the gut microbiota diversity and has positive effects on the preservation of the bone composition and memory function, the exact mechanisms remain to be further explored. Future investigation will study the effects of fecal implantation of vehicle- or FLL-treated aged mice into FLL- or untreated- mice, respectively, on bone quality and cognition functions. In addition, administration of FLL to aging mice prevents an elevation of TMAO production and oxidative stress, which subsequent promotes Sirt6 expression in the current study. FLL has been demonstrated to exhibit antioxidant activity in OVX rats [10], in carbon tetrachloride stimulated rats [60], and in acute butylated hydroxytoluene stimulated rats [61], as well as in SH-SY5Y cells [62]. The enhanced oxidative stress results in an inhibition of Sirt6 expression [13, 47]. However, further observation on the effects of FLL on Sirt6 expression in response to chronic injection of TMAO is still needed, which will provide strong evidence on underlying mechanisms of this herb on senile osteoporosis. Nonetheless, the present study provides the first evidence that FLL may protect against senile osteoporosis through regulation of gut microbiota diversity and the levels of TMAO and Sirt6, and therefore could be a new source for screening anti-aging drug candidates. Clinically, accumulating evidence also suggests that FLL is effective in protection against aging [63].

## CONCLUSION

**Figure 8:**
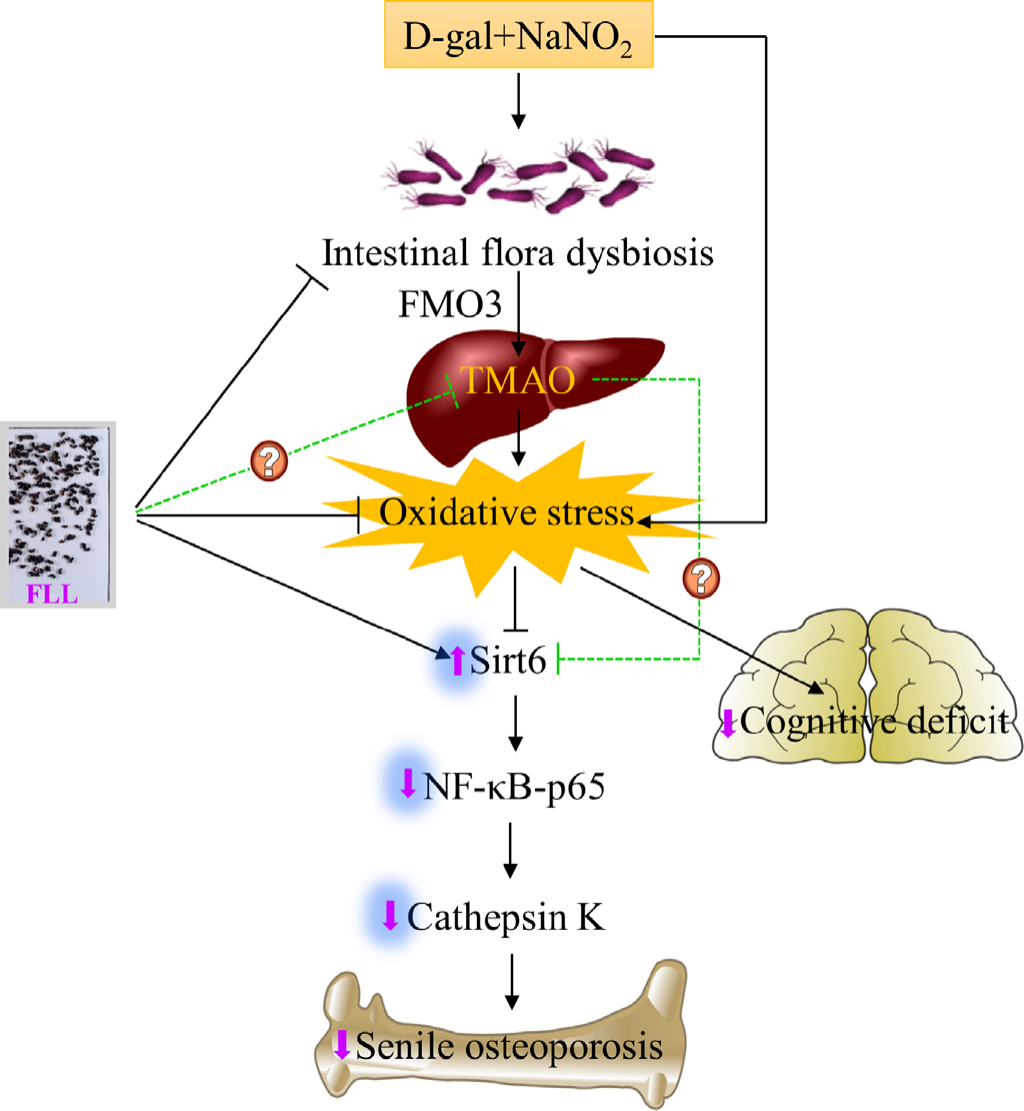
**Schematic diagram illustrating the underlying mechanism of D-gal and NaNO_2_ on inducing senile osteoporosis in mice and the action of *Fructus Ligustri Lucidi* (FLL) on it.** The injection of D-gal and NaNO_2_ triggers gut dysbiosis and cognition impairment followed by upregulation of circulated TMAO levels through enhancement of FMO3 expression in the liver, which further results in compromised bone quality and cognition via an increase of oxidative stress, downregulation of Sirt6, and the activation of NF-κB/CatK signaling. Administration of FLL to aging mice exerts a bone protective effect by increasing Sirt6 expression and inhibiting the NF-κB/CatK signaling through the regulation of gut microbiota composition, which contributes to the downregulation of TMA and TMAO production, and the improvement of antioxidant activity, and the subsequent increase of Sirt6 expression. However, whether FLL could directly regulate TMAO production in this process still remains unexploited. The arrow sign (↑) indicates promoting. The stop sign (┴) indicates inhibiting. The green dotted line indicates unconfirmed action.

In summary, we are the first to demonstrate that the acceleration of senescence caused by administration of D-gal and NaNO_2_ to ICR mice exhibited an osteoporotic bone phenotype. This was associated with aggravated oxidative stress and activated Sirt6/NF-κB/CatK signaling and the disruption of gut microbiota, including increased levels of FMO3 and TMAO (Figure 8). Remarkably, FLL aqueous extract may preserve the aging-related bone quality through inhibiting the above-mentioned signaling pathways. Therefore, FLL could be considered as a source for identifying drug candidates in the treatment of senile osteoporosis. However, whether FLL could directly influence TMAO levels still remains unexplained. Additional studies are required to elucidate the active ingredients in FLL aqueous extracts that exert the bone- and cognition-protective activities.

## MATERIALS AND METHODS

### Chemicals and antibodies

D-gal (Cat# G0750), NaNO_2_ (Cat# 31443), and TMAO (Cat# 317594) were purchased from Sigma-Aldrich (St. Louis, MO, USA). Enzyme-linked immunosorbent assays (ELISA) kits used for determination of TNF-α, IL-2, and 8-OH-dG were purchased from Beijing Fangcheng Biotechnology Company (Beijing, China). MDA, TAC, GSH, and GSSG kits were purchased from Nanjing Jiancheng Bio-Engineering Institute (Nanjing, China). P1NP and CTx-1 were obtained from CUSABIO (Wuhan, China). Deuterated trimethylamine-N-oxide (d9-TMAO) was purchased from BioRuler (Cat#: RH141121; Connecticut, USA). Antibodies against Sirt6 (Cat# ab62739), CatK (Cat# ab19027), FMO3 (Cat# ab126711), NF-κB-p65 (Cat# ab16502), and NF-kB-pp65 (Cat# ab86299) were procured from Abcam (Cambridge, MA, USA). Antibody against Nox4 (Cat# NB110-58849) was obtained from Novus Biologicals (Littleton, CO, USA). The antibody against acetyl-NF-κB p65 (Cat# YK0018) was purchased from ImmunoWay Biotechnology Company (Plano, TX, USA).

### Preparation of FLL aqueous extract

FLL aqueous extract was prepared as described previously [10]. Briefly, FLL was bought from Beijing TongRenTang pharmacy (Beijing, China) and certified by Professor Zexin Ma (TCM museum at Beijing University of Chinese Medicine). Wine-steamed (processing the pieces of FLL under the method for steaming with wine until the drug darkens thoroughly) FLL (100 g) was grinded into powder and dissolved in distilled water (1000 ml) by continuously stirring for 48 h at 4 °C. Then the supernatants were harvested by centrifugation (5000 rpm at 4 °C for 5 min), and lyophilized to obtain a powder (20g, 1 g contains 5 g wine-steamed FLL).

### Animals and induction of aging models

Male ICR mice (20 ± 2 g) were purchased from China SiBeiFu Biotechnology Co. Ltd. [Beijing, China; certification No: SCXK (Jing) 2017-0002], and housed in specific-pathogen-free (SPF) clean level conditions [certification number SYXK (Jing) 2017-0038] with controlled temperature (22 ± 1 °C), humidity (50 ± 5%), a 12-h light/dark cycle, and free access to food and water. Our protocols were followed the guidelines established by the BUCM Animal Care Committee.

After 1 week of acclimation, the mice were randomly divided into the following four groups (each containing 10 animals): Normal group, Aging group, Aging+VE group, and Aging+FLL group. Mice in the last three groups were daily intraperitoneally injected with D-gal (120 mg/kg/day) and NaNO_2_ (90 mg/kg/day) in 0.9% sterile saline for 3 months [64] to induce the premature aging process. Meanwhile, mice in the Normal group were daily intraperitoneally injected with the same volume of 0.9% sterile saline. Commencing on the 25^th^ day, the mice in the groups of Aging+VE and Aging+FLL received intragastric administration of VE (100 mg/kg/day; dissolved in 0.1% Tween 80) or FLL aqueous extract (4.9 g/kg/day; dissolved in 0.1% Tween 80) for 65 days. Mice in the Normal and Aging groups received oral administration of the same volume of 0.1% Tween 80.

### Morris water maze test

After 65 days of treatment, all mice were subjected to the water maze task as reported previously, with slight modifications [65]. Briefly, the experimental apparatus was composed of a circular, black pool (100 cm in diameter, 35 cm in height) filled with water (23 ± 1 °C) containing a black, edible pigment. Beyond that, the set-up also included a camera and corresponding image analysis software. The swimming pool was divided into four equal quadrants (I-IV) and an escape platform was submerged 1 cm below the water surface in the midpoint of the IV (southeast IV) quadrant. For memory training purposes, each mouse was given 1 minute to find the hidden platform after being put into the water. Once mice located the platform, it was allowed to remain on it for 10 s. If they failed to find the platform within 1 minute, they were put on the platform for 15 s and the latency to the platform was recorded as 1 minute. Each day they were successively put into the water from three directions (southwest I, northwest II, northeast III) and continuously trained for 4 days. On the fifth day, the swimming traces of the mice were recorded (after removing the platform) and data were analyzed using the TopScanLite-TopView Behavior analyzing system (Noldus Information Technology, Leesburg, VA, USA). Subsequently, the time needed to reach the apparent platform area, the time spent in each quadrant, and the number of times the mice crossed the platform position (numbers of crossing) were recorded and calculated for statistical analysis.

### Tissue and organ collection

Three days after the water maze test, mice were anesthetized with 1% sodium pentobarbital. Then, stool samples were collected from the colon and immediately soaked in liquid nitrogen. Blood was harvested from the orbital sinus and serum prepared by centrifugation (3,000 rpm for 15 min). Tibias, femurs, and livers were collected from the mice after cervical dislocation. All samples were either stored at -80 °C or fixed in formalin for further analysis.

### Serum biomarkers determination

The serum levels of 8-OH-dG, MDA, TAC, GSH, GSSG, TNF-α, IL-2, P1NP and CTx-1 were determined by appropriate kits following the manufacturer’s instructions.

### LC/MS/MS analysis

Circulatory TMAO levels were determined by LC/MS/MS as previously described, with slight improvements [27, 66, 67]. Briefly, serum samples (30 μl) were mixed with 90 μl of internal standard solution (10 μM d9-TMAO in acetonitrile) in 1.5 ml Eppendorf tubes. Then, the mixture was immediately centrifuged (13,200 rpm at 4 °C for 15 min). Subsequently, 90 μl of the supernatants were injected into an ultimate silica column (2.1 × 100 mm, 5 μm, Welch Materials, Inc.; Cat. No. 00200-31012; Shanghai, China) at a flow rate of 0.3 ml/min using an Agilent Technologies 1200 series pump system, and an Agilent 6410A Triple Quad mass spectrometer (California, USA). The analytes were eluted by mixing solvent A (acetonitrile) with solvent B (10 mM ammonium formate in water) at a ratio of 70% A:30% B. Electrospray ionization in positive-ion multiple reaction monitoring mode was used to monitor the analytes. The characteristic production transitions of TMAO and d9-TMAO are at m/z 76→58 and m/z 85→66, respectively. For the precise quantitative analysis, a standard curve (0-100 μM) was generated as mentioned above (R^2^>0.99).

### μCT analysis

Right femurs were analyzed by μCT (PerkinElmer Quantum GX-micro-CT) as previously described, with some modification [8]. Briefly, the sequential trans-axial images of the distal femurs (13 mm) were obtained using an isotropic voxel size of 13 μm, current of 88 μA, scanning time of 14 min, and peak tube voltage of 90 kV. The scanning angular rotation was 360 degrees. Data sets were reconstructed using the provided software (PerkinElmer Quantum self-software). To analyze the bone micro-architectural properties, volumes of interest of the femur were selected starting at 0.5 mm (40 image slices) from the growth plate and extending a longitudinal distance of 2.0 mm (140 image slices) in the proximal direction and subsequently evaluated (Analyze 12.0 software). The following parameters were obtained: TV, BV, BV/TV, Tb.N, Tb.Th, Tb.Sp, Tt.Ar inside the periosteal envelope, Ct.Ar, and average Ct.Th.

### Bone biomechanical strength assay

After μCT scanning, right femurs were submitted to a three-point bending test using an electronic universal testing machine (Shimadzu Corporation, AGS-X500, Japan), as previously described [68]. In brief, the shaft of the femur was fixed between two supporting points, with a distance of 9 mm. A load was vertically applied to the femoral mid-shaft, with a displacement speed of 1mm/min until the femoral shaft fractured. The ultimate load, bending strength, and elastic modulus of the femurs were determined by TRAPEZIUM LITE X 1.0.2b software.

### FTIR

Bone material properties were determined using FTIR as previously described [8]. Briefly, the right femurs were ground into powder in a ceramic mortar under liquid nitrogen. After that, 1% (w/w) of the dried femoral powders were mixed with potassium bromide (KBr) and pressed into an optically transparent tablet (10 tons, 30 s). The KBr tablet was scanned using FTIR (PerkinEImer, America) in transmission mode at 4 cm^−1^ resolutions in the 4,000 – 400 cm^−1^ range, accumulating 64 scans. Finally, collagen maturity (collagen cross-link ratio) was determined as the relative intensity ratio of 1,660 to 1,690 cm^−1^. The levels of mineralization (mineral/matrix) were assessed as the area of the phosphate band (~900–1,200 cm^−1^) divided by the area of the amide I band (~1,595–1,860 cm^−1^). The degree of carbonate substitution into the hydroxyapatite crystal was determined by the relative peak area ratio between the carbonate (~850–890 cm^−1^) and phosphate bands (~900–1,200 cm^−1^). Crystallinity was defined as an intensity ratio of 1030 and 1020 cm^−1^. The acid phosphate substitution was assessed as an intensity ratio of 1128 and 1096 cm^−1^ according to the second derivative spectrum.

### H&E and safranin O/fast green staining of tissue samples

Right femurs from the mice were immersed in 4% paraformaldehyde for 72 h and then decalcified in 5% nitric acid for 1 week. The decalcified femurs were dehydrated and defatted with graded ethanol (50% – 100%) and xylene and then embedded in paraffin. Sections of 4-μm thickness were used for H&E and safranin O/fast green staining. H&E staining was performed by following routine procedures [69].

Safranin O/fast green staining was performed as previously described [70]. Briefly, the dehydrated slides were stained for 5 min with hematoxylin solution and differentiated with 1% HCl-EtOH for 2 s, and then sequentially stained for 5 min each with 0.05% fast green and 0.1% safranin O.

The areas of the mounted sections were evaluated and photographed using an Olympus BX53 fluorescence microscope (Tokyo, Japan). The relative Integrated Optical Density (IOD) of safranin O/fast green staining were analyzed using Image-Pro Plus 6.0 software.

### IHC staining

The IHC staining was conducted as described [68]. Briefly, 5-μm longitudinal sections of paraffin-embedded femurs were de-paraffinized with xylene and hydrated with an ethanol gradient series (100%-70%). The sections were then incubated in antigen retrieval solution, followed by overnight incubation at 4 °C with primary antibodies [Sirt6 (1:200), CatK (1:100), acetyl-NF-κB-p65 (Lys310) (1:100)], and finally by incubation with the appropriate horseradish peroxidase-conjugated secondary antibodies at room temperature. After diaminobenzidine and hematoxylin treatment, sections were photographed using an Olympus BX53 fluorescence microscope. The intensity of the positive staining (brown particles) was analyzed using Image-Pro Plus 6.0 software and expressed as an IOD value.

### Western blot analysis

Western blot was conducted according to a previously described protocol [70]. Briefly, the membrane was sequentially incubated with the appropriate primary antibody [Nox4 (1:500), FMO3 (1:1000), Sirt6 (1:1000), NF-κB-p65 (1:500), NF-κB-pp65 (1:500), and CatK (1:500)], and then with the corresponding horseradish peroxidase-conjugated secondary antibody. Afterwards, immunopositive bands were visualized by hypersensitive electrochemiluminescence (ECL) liquid and the images captured by an Azure Bio-imaging system (Azure C500; California, USA). The grey values of the bands were quantified with the Image-Pro Plus 6.0 software and normalized with the same membrane of β-actin (1:2000) blots as the internal control.

### Fecal DNA extraction and 16S ribosomal DNA sequencing

Total microbial genomic DNA was isolated from stool samples using the DNeasy PowerSoil Kit (QIAGEN, Inc., Netherlands) according to the manufacturer’s instructions. PCR amplification was performed as follows [71]. Briefly, V3–V4 regions of bacterial 16S rRNA genes were amplified using the forward primer 515F (5′-GTGCCAGCMGCCGCGGTAA-3′) and the reverse primer 907R (5′-CCGTCAATTCMTTTRAGTTT-3′). A single-step PCR was done under the following conditions: 98 °C for 2 min, followed by 25 cycles of 98 °C for 15 s; 55 °C for 30 s and 72 °C for 30 s; and at 72 °C for 5 min for a final extension. The PCR amplicon products were then purified with Agencourt AMPure Beads (Beckman Coulter, Indianapolis, IN, USA) and quantified using a PicoGreen dsDNA Assay Kit (Invitrogen; Carlsbad, CA, USA). Finally, amplicons were pooled in equal amounts, and paired-end 2,300 bp sequencing was performed utilizing the Illumina MiSeq platform and MiSeq Reagent Kit.

### Bioinformatics and statistics

Sequencing data of the gut microbiota were processed using the Quantitative Insights Into Microbial Ecology (QIIME, v1.8.0) pipeline [73]. Specifically, raw sequencing reads were assigned to respective samples and identified as valid sequences. The low-quality sequences were filtered through the following criteria [72]: short sequences < 150 bp, sequences with average Phred quality scores of < 20, sequences with ambiguous bases, and sequences with mononucleotide repeats of > 8 bp. The remaining sequences were clustered into OTUs at 97% sequence identity by UCLUST. OTU taxonomic classification was conducted by BLAST searching the representative sequences set against the Greengenes Database using the best hit [73]. An OTU table was further generated to record the abundance of each OTU in each sample and the taxonomy of these OTUs. OTUs with less than 0.001% of total sequences in all samples were discarded. To minimize the difference of sequencing depth among samples, an averaged and rounded rarefied OTU table was generated by averaging 100 evenly resampled OTU subsets under the 90% of the minimum sequencing depth for further analysis.

Sequence data analyses were mainly performed using QIIME and R packages (v3.2.0). OTU-level alpha diversity indices, such as the Chao1 richness estimator, Abundance-based Coverage Estimator metric, Shannon diversity index, and Simpson index, were calculated using the OTU table in QIIME. Linear discriminant analysis effect size LEfSe was performed to detect differentially abundant taxa across groups using the default parameters [74].

### Statistical analysis

All data obtained from animal experiments were expressed as the mean and standard deviation $\left(\bar{x}\pm \text{SD}\right)$. When the data met a normal distribution and variances were homogeneous, the one-way analysis of variance (ANOVA) test was used (Version 22.0, IBM SPSS Statistics, IBM Corp., Armonk, New York, NY, USA). When the data met a normal distribution but the variances were not homogeneous, the Dunnett’s T3 test was used. In cases where the data did not meet a normal distribution, nonparametric analysis was employed. Statistical significance was determined at *p* < 0.05.
